# Frequency of Meningitis in Children Presenting with Febrile Seizures at Ali-Asghar Children’s Hospital

**Published:** 2014

**Authors:** Azita TAVASOLI, Ladan AFSHARKHAS, Abdolmajid EDRAKI

**Affiliations:** 1 Pediatric Neurology Department, Ali-Asghar Children’s Hospital, Iran University of Medical Sciences, Tehran, Iran.; 2 Pediatric Department, Ali-Asghar Children’s Hospital, Iran University of Medical Sciences, Tehran, Iran.

**Keywords:** Bacterial meningitis, Febrile seizure, Meningitis

## Abstract

**Objective:**

Febrile seizures (FS) are the most common type of childhood seizures, affecting 2–5% of children. As the seizure may be the sole presentation of bacterial meningitis in febrile infants, it is mandatory to exclude underlying meningitis in children presenting with fever and seizure. To determine the frequency of meningitis in children with FS and related risk factors, the present study was conducted at Ali-Asghar Children’s Hospital.

**Materials & Methods:**

The records of children aged from 1-month–6 years of age with fever and seizure admitted to the hospital from October 2000–2010 were studied. The charts of patients who had undergone a lumbar puncture were studied and cases of meningitis were selected. The related data was collected and analyzed with SPSS version 16.

**Results:**

A total of 681 patients with FS were known from which 422 (62%) lumbar punctures (LP) were done. Meningitis (bacterial or aseptic) was identified in 19 cases (4.5%, 95% CI 2.9–6.9 by Wilson- Score internal) and bacterial meningitis in 7 (1.65%, 95% CI 0.8–3.3). None of the patients with bacterial meningitis had meningeal irritation signs. Complex FS, first attack of FS, and impaired consciousness were more common in patients with meningitis when compared to non- meningitis patients.

**Conclusion:**

Meningitis is more common in patients less than 18 months presenting with FS; however, complex features of seizures, first attack of FS, or impaired consciousness seem significant risk factors for meningitis in these children and an LP should be considered in this situation.

## Introduction

Febrile seizures (FS) are the most common type of childhood seizures, affecting 2–5% of children older than 1 month and most commonly from 6 months–5 years old (1,2). It is possibly a major cause of pediatric admissions worldwide (3). FS are defined as seizures accompanied by fever without evidence of intracranial infection, metabolic disturbance, or a history of previous afebrile seizures (4).

They are categorized as either simple (generalized, lasting less than 15 minutes and occurring only once in a 24-hour period) or complex (focal seizure, lasting more than 15 minutes or recurrent in a 24-hour period) (4).

Although FS induce by age related hyper-excitability of the brain to fever, determining the cause of the fever is critical in the evaluation of these children (5). The relationship between seizure and bacterial meningitis has been identified well in previous studies (6,7). Seizures may be the sole presentation of bacterial meningitis in febrile infants (8). Seizures are the first manifestation of meningitis in 16.7% of children and in one-third of these patients, whereas meningeal signs and symptoms may not be evident (5). Therefore, it is mandatory to exclude underlying meningitis in children presenting with fever and seizure prior to making the diagnosis of FS (9). The initial concern in these children is always to make a proper decision regarding to do an LP to exclude meningitis. Awareness of the prevalence of meningitis and its related factors in children presenting with FS to help physicians to make proper decisions in these situations is necessary (9). There are several clinical studies worldwide reporting the prevalence of meningitis among children with FS (10-13). Some of these studies suggest that in the absence of typical meningeal signs, then an LP should be considered in children with complex seizures, prior antibiotic therapy, age less than 12 months, or incomplete vaccination history (14).The present study determines the prevalence of meningitis and its associated risk factors in children with FS referred to Ali-Asghar Children’s Hospital.

## Materials & Methods

In this retrospective cross-sectional study, we included all consecutive children aged 1 month to 6 years who presented at Ali-Asghar Children’s Hospital with a complaint of fever and seizure from October 2000–2010 and used the medical records as our data. Exclusion criteria included history of previous non-febrile seizures or a previously diagnosed underlying illness associated with seizures, an immune compromised state, or the presence of a ventriculoperitoneal shunt or known trauma. The data collected were age, gender, type of seizure, history of previous FS, signs of meningeal irritation (neck stiffness, Kernig’s or Brudzinski’s Sign(s)), impaired consciousness lasting longer than one hour after seizure, pretreatment with antibiotics (from any rout for days before occurring seizure), and results of LP, cerebrospinal fluid (CSF) culture, and blood culture. CSF pleocytosis was defined as a white blood cell (WBC) count of 6μL or more in the CSF (15). The WBC count for traumatized LP was determined by using the following corrections: corrected CSF WBC count= (CSF WBC count – [CSF red blood cell count/500]) (1). Bacterial meningitis (BM) was defined as growth of a pathogen from CSF culture or CSF pleocytosis with growth of a pathogen from the blood culture. CSF pleocytosis with mononuclear cell dominancy and no growth of a pathogen from CSF or blood culture was considered as aseptic meningitis if the patient was not pretreated with antibiotics during the previous week. In cases with CSF pleocytosis and history of pretreatment with antibiotics if a diagnosis of BM was given on the side of caution and the patient was treated as having BM, we identified her or him as a case of BM. 

Data were analyzed using SPSS version 16. A Fisher’s Exact test was used for statistical analysis and a p<0.005 was considered significant.

## Results

A total of 681 patients with a diagnosis of FS were identified. An LP was performed in 422 patients (62%). In 403 patients with mean age of 26±2.7 months, an LP was normal. Of those 217 patients were male (54%) and 186 were female (46%). In children who had not undergone an LP, meningitis could be excluded according to clinical status. In the follow-up visits, none of them subsequently had returned to the hospital with a diagnosis of meningitis. Diagnosis of meningitis (bacterial or aseptic) was identified in 19 cases (4.5%, 95% CI 2.9–6.9). The mean age of the patients with meningitis was 10±3.2 months including 11 males (58%). 

Seven patient met the study criteria for BM (1.65%, 95% CI 0.8–3.3). Organisms grown were Hemophilus Influenza in 2 cases and Streptococus Pneumoniae in 3 cases from the CSF cultures and gram positive Streptococcus in 2 cases from the blood cultures. The remaining 12 patients with CSF pleocytosis had aseptic meningitis. The majority of patients with meningitis and all patients with BM were in the age group less than 18 months old. Among patients who had a normal LP, 106 cases (26.3%) presented with complex and 297 (74.7%) with simple FS while complex FS was seen in 16 patients (84.2%) with meningitis (bacterial or aseptic) and this was statistically significant (p <0.001). All patients with BM had complex FS. Impaired consciousness was seen in 15 cases with meningitis (78.9%) and 6 cases with bacterial meningitis (85.7%) compared with 26 cases with a normal LP (6.5%) and this was statistically significant (p <0.001). None of the patients with BM had meningeal irritation signs. Neck stiffness was present in 2 patients with aseptic meningitis and both were older than 5 years of age. The majority of patients with meningitis had presented with their first attack of FS (17cases, 89.5%) when compared with patients with a normal LP (45.2%) and this was statistically significant (p<0.001). All patients with BM had presented with their first attack of FS. Six patients with meningitis (31.6%) and 136 patients with a normal LP (33.7%) were been pretreated with antibiotics before admission and this was not statistically significant. Table 1 illustrates the comprehensive data regarding the characteristics of patients.

## Discussion

The correlation between seizures and meningitis is well known and has been reported in 12–27% of cases (1). Although, the possibility of BM in a febrile child has been reduced since the introduction of universal immunization against bacterial pathogens (1), this is still a major concern in any child presenting with FS especially in populations with lower immunization rates. The results of our study show a 4.5% frequency of meningitis (bacterial and aseptic) and 1.6% of BM in children with FS. These results are similar to those of Ehsanipour et al that reported 3.6% of meningitis and 1.6% of BM in children aged 6 mo.–5 yr. with FS (16). 

In addition, Al-Eissa in his study in Saudi Arabia found a 3.5% frequency of meningitis and 1.5% of BM among patients aged 3mo-5 yr. with FS (17). Casasoprana et al (2013) indicated that in France the frequency of BM was 1.9% in children aged less than 18 months with a first FS (14). Ghotbi and Shiva showed 4.7% meningitis among 254 cases with seizure and fever (18). Some studies have reported higher rates of meningitis among children with FS. Owusu-Ofori et al found 10.2% rate of BM among children with FS in China (10) and similarly Tinsa et al from Tunisia reported 10% BM in children aged less than one year with FS (19). This could be due to a higher prevalence of meningitis in these populations (10). Joshi Batajoo et al in Nepal reported 17% meningitis and 4.5% BM in children with the first episode of fever and seizure (20). These results may be partly due to studies of children with the first episode of FS that could be more associated with meningitis. 

On the other hand, the results of some studies are quite different. Caroll and Brookfield have reported that 0.23% rate of BM in children with simple FS, after the implementation of universal immunization (21). Kimia et al reported the rate of 0.9% of BM among children with complex FS (1). They reported no cases of BM in another study on children aged 6–18 months with the first attack of simple FS (11). Shaked et al found similar results for 6–12 month old children with the first simple FS (12). The results could be related to a higher coverage area of vaccination against common bacterial pathogens in childhood meningitis in these populations. 

We did not find any significant correlation between gender and meningitis. This is in parallel with other studies (10,16,18). Numerous studies on FS have reported that meningitis is more common the younger the child (10,14,17,18,20). While, in our study, meningitis was more prevalent in children less than 18 month old, but this was not statistically significant. This may be due to an LP not done for all patients with FS and the many patients in whom an LP was not done were older. Additionally, the majority of other studies have included children up to 5 years of age but our study was conducted on a wider age range. Similar to Joshi-Batajoo (20) and Laditan AA (22), none of our patients with BM had meningeal irritation signs, further indicating that the lack of these signs does not exclude meningitis in younger patients. Physicians should not rely solely on physical signs in this regard. Our results showed the majority of children with meningitis had complex seizure features. 

This is comparable with the results of Ham and Medwid (2) and Casasoprana (14) that found the possibility of BM or encephalitis is very low in children with simple FS and a normal physical examination. Rossi et al reported that in children less than 3 years old with an absence of neurological signs, a complex seizure could be an important distinguishing factor between patients with and without meningitis (23). Najaf-zadeh et al reported a 0.6% prevalence of BM among children with complex versus 0.2% in simple FS (8). Al-Eissa reported all patients with meningitis had complex seizure features (17). Other studies have reported similar results (2,14,16,18,20). While Kimia et al reported a low frequency of BM in children with the first complex FS, the majority of patients with meningitis, and all with BM in our study had presented with their first attack of FS when compared with non-meningitis patients. Our results are parallel with of the Ehsanipour et al (16). Children with a genetic predisposition may experience recurrent attacks of FS in childhood (24). Besides, seizure as a presenting sign of meningitis is seen in almost 25% of young children (4). Therefore, in all febrile patients with seizures, the lack of history of previous FS may lead to more suspicion of the involvement of meningitis. 

In our study, patients with meningitis had significantly greater frequency of impaired consciousness. Some other studies have reported similar results (14,17,18). In comparison, Owusu-Ofori reported 4.5% rate of BM in patients presented with FS and lethargy in comparison with 34.6%in patients with FS and neck stiffness (10). 

This could be due to age of the patients the study was conducted on, up to 15 years, which in this age group meningeal signs with the inclusion of neck stiffness are more evident. There was not any significant correlation between pretreatment with antibiotics and frequency of meningitis in our patients. Ghotbi and Shiva found that prior antibiotic use is a risk factor for meningitis among children with FS (18). Casasoprona et al concluded that in cases of prior antibiotic therapy in children with FS, an LP should be considered (14). These results are different with ours, which could be due to inappropriate increased use of antibiotics in febrile children in our community. According to the recent guidelines by the American Academy of Pediatrics, an LP is an option in the evaluation of children presenting with seizure and fever that is pretreated with antibiotics as it can mask the signs and symptoms of meningitis. Indeed the decision to perform an LP depends on the type and duration of the antibiotic used and left to the discretion of the clinician (4).

There are few limitations to our study. An LP was not done for all patients and some patients with aseptic

meningitis (without significant clinical evidence) could be missed. So, we mentioned in our results a meningitis frequency based on cases in which LP was done (19 of 422 patients [4.5% CI 2.9–6.9]) instead of 19 of 681 for all patients (2.79% [95% CI 1.7-4.3]). 

**In conclusion,** our findings suggest that the frequency of bacterial meningitis in children who presented with FS is not high. Meningitis is more common in patients less than 18 months old with FS; however, complex features of seizures, first attack of FS or impaired consciousness seem to be significant risk factors for meningitis and an LP should be in mind in this situation. Lack of meningeal irritation signs does not exclude meningitis especially in younger children.

**Table 1:** The characteristics Compared between Patients Presenting with FS With and Without Meningitis

**Variables**	**Meningitis (n=19)** **No. (%)**	**Non-meningitis (n=403)** **No. (%)**	**Odds Ratio**		**95% CI**	**P-value** ^*^
**Gender**						
Female	8 (42.1%)	186 (46.2%)	0.848	0.334-2.154		0.816
Male	11(57.9%)	217 (53.8%)				
**Age**						
18 m**.**	15 (78.9%)	190 (47.1%)	4.204	1.371-12.886		00.9
> 18 m**.**	4 (21.1%)	213 (52.9%)				
**Seizure type**						
Simple	3 (15. 8%)	297 (73.7%)	0.067	0.019-0.234		<0.001
Complex	16 (84.2%)	106 (26.3%)				
**Seizure recurrence**						
First attack	17 (89.5%)	182 (45.2%)	10.321	2.354-45.262		<0.001
Recurrent attack	2 (10.5%)	221 (54.8%)				
**Altered consciousness**						
No	4 (21.9%)	377 (93.5%)	54.375	16.84-175.61		<0.001
Yes	15 (78.9%)	26 (6.5%)				
**Pre-treatment with antibiotic**						
No	13 (68.4%)	267 (66.3%)	0.906	0.337-2.436		1.00
Yes	6 (31.6%)	136 (33.7%)				

Fisher’s Exact Test
